# Left Ventricular Thrombus Formation in Acute Anterior Wall Myocardial Infarction: A Comparison Between Thrombolyzed and Non-Thrombolyzed Patients

**DOI:** 10.7759/cureus.9090

**Published:** 2020-07-09

**Authors:** Kashif A Hashmi, Hadi Y Saeed, Jawad Ahmed, Javeria Najam, Muhammad Irfan, Atif A Hashmi

**Affiliations:** 1 Cardiology, Chaudhry Pervaiz Elahi Institute of Cardiology, Multan, PAK; 2 Medicine, Liaquat National Hospital and Medical College, Karachi, PAK; 3 Statistics, Liaquat National Hospital and Medical College, Karachi, PAK; 4 Pathology, Liaquat National Hospital and Medical College, Karachi, PAK

**Keywords:** left ventricular thrombosis, anterior wall acute myocardial infarction, thrombolytic therapy

## Abstract

Introduction

Left ventricular thrombus (LVT) formation is a prominent complication of acute myocardial infarction (AMI). Accurate and prompt detection of the condition is important as it poses a high risk for thromboembolic events that can be arrested by systemic anticoagulation. The purpose of our study was to evaluate the frequency of LVT formation in thrombolyzed and non-thrombolyzed patients with AMI to ascertain the current magnitude of the problem in the local population.

Methods

The study was conducted at the Chaudhry Pervaiz Elahi Institute of Cardiology in Multan, Pakistan. A total of 281 patients of either gender aged between 30-65 years with anterior wall myocardial infarction (AWMI; both thrombolyzed and non-thrombolyzed) were included in the study. Once they were enrolled in the study, all the relevant baseline investigations were performed. A detailed history was taken and examinations were done; serial ECG and echocardiography were performed till discharge from the hospital on the third day of hospitalization to record the final outcome of the study, i.e., LVT formation.

Results

The mean age of the patients was 55.54 ± 7.26 years. Overall, LVT formation was noted in 65 cases (23.1%), of which 11 (16.9%) were thrombolyzed patients and 54 (83.1.1%) were non-thrombolyzed. A significant association of LVT was noted with age, hypertension, family history, and duration of symptoms.

Conclusion

We found a high frequency of LVT formation among patients with AWMI who have not undergone thrombolytic therapy. It was observed that LVT was notably associated with advanced age, hypertension, and other comorbidities. Early presentation to the hospital and thrombolysis reduce the risk of developing LVT, which in turn can reduce morbidity and mortality in such patients.

## Introduction

Ischemic heart disease (IHD) is a major cause of morbidity and mortality worldwide [1-3]. Males are more commonly affected by this condition than females. Atherosclerotic coronary artery disease (CAD) causing myocardial ischemia may manifest itself either as acute myocardial infarction (AMI), unstable angina, or effort angina. Among these, AMI is the most predominant life-threatening disease usually encountered in emergency hospital admissions. The mortality rate for AMI has decreased by about 30% over the last two decades, but it still remains high, especially in underdeveloped countries. The cardiovascular comorbidities associated with AMI are on the rise in Pakistan where 18% of the adult population suffers from hypertension, tobacco addiction, and obesity.

Left ventricular thrombus (LVT) is a blood clot that forms in the left ventricle of heart due to stasis of blood. LVT leading to cerebrovascular accidents is a potentially catastrophic complication following anterior ST-segment elevation myocardial infarction (STEMI) [4]. Although anticoagulation is often prescribed for this, data regarding the appropriate drug, duration, risks, and effect on echocardiographic indices of thrombus are lacking. Moreover, given the difficulty in obtaining adequate anticoagulation with warfarin, it is possible that short-term treatment with a more predictable agent would be more effective [5,6]. Acute thromboembolic events due to LVT formation, particularly in patients with reduced ejection fraction (EF), remain a risk for patients surviving anterior STEMI. The reported incidence of LVT formation and subsequent embolization varies based on the timing of the echocardiographic examination and the diagnosis, anticoagulation, and reperfusion strategies utilized in managing the initial presentation. However, it is estimated to range from 0 to as high as 86% [7,8]. A study conducted by Zia et al. has reported LVT formation in 15.4% of patients with anterior wall acute myocardial infarction (AWMI). While LVT was found in 5.4% of the patients thrombolyzed with streptokinase, it was present in 30.4% of those who were not thrombolyzed [9].

The purpose of our study was to evaluate the frequency of LVT formation in thrombolyzed and non-thrombolyzed patients with AMI to ascertain the current magnitude of the problem in the local population. We believe that the results of this study will help clinicians to anticipate LVT among the targeted population and treat them accordingly. We also feel that the study provides useful baseline information on this topic and highlights the importance of thrombolytic therapy in AMI patients, which will contribute to improving clinical outcomes in these patients. Finally, we think our findings will improve the quality of life of the patients and will also help reduce hospital costs, which will be beneficial for national health economies.

## Materials and methods

This descriptive study was conducted at the Department of Cardiology, Chaudhry Pervaiz Elahi Institute of Cardiology in Multan, Pakistan. We conducted the study after obtaining approval from the institute's Ethical Review Committee. A total of 281 patients of either gender aged between 30-65 years with AWMI (both thrombolyzed and non-thrombolyzed) were included in the study based on consecutive sampling over a period of one year. Informed consent was taken from the patients/attendants of patients after explaining the objectives of this study, ensuring the confidentiality of the information provided, and providing assurance that the patients were in no danger due to participating in this research. Once they were enrolled in the study, all the relevant baseline investigations were performed. A detailed history was taken and examinations were done; serial ECG and echocardiography were performed till discharge from the hospital on the third day of hospitalization to record the final outcome of the study, i.e., LVT formation. All the data was recorded on a predesigned proforma.

Statistical analysis

All the data was entered and analyzed using IBM SPSS Statistics for Windows, Version 20.0 (IBM, Armonk, NY). Descriptive statistics were applied to calculate the mean and standard deviation for the age of the patients and the duration of symptoms. Frequencies and percentages were compiled for categorical variables. The frequency of LVT was compared between thrombolyzed and non-thrombolyzed patients by applying the chi-square test at a level of significance of 0.05. Effect modifiers like age and diabetes were controlled by preparing stratified charts. The post-stratification chi-square test was applied to see its effect on outcomes. A p-value of ≤0.05 was considered to be statistically significant.

## Results

Our study comprised a total of 281 patients who met the inclusion criteria. Of the 281 patients, 184 (65.5%) were male and 97 (34.5%) were female. The mean age of the patients was 55.54 ± 7.26 years (range: 40-65 years). Most of the patients (204, 72.6%) were more than 50 years of age. Of the 281 patients, 106 (37.7%) belonged to rural areas and 175 (62.3%) belonged to urban areas. Diabetes was present in 87 (31%) subjects, hypertension in 130 (46.3%), and 65 (23.1%) had a history of smoking. The mean duration of symptoms was 7.25 ± 3.82 hours, and 151 (53.7%) patients had a duration of symptoms for more than six hours. Family history was positive in 106 (37.7%), while thrombolytic therapy was given for 194 (69.0%) of our study cases. LVT formation was noted in 65 (23.1%) cases, of which 11 (16.9%) were thrombolyzed patients and 54 (83.1%) non-thrombolyzed. The characteristics of the study population are presented in Table 1.

**Table 1:** Characteristics of the study population

Characteristic	N (%)
Gender	
Male	184 (65.5)
Female	97 (34.5)
Age group	
≤50 years	77 (27.4)
>50 years	204 (72.6)
Residence	
Rural	106 (37.7)
Urban	175 (62.3)
Diabetes mellitus	
Present	87 (31)
Absent	194 (69)
Hypertension	
Present	130 (46.3)
Absent	151 (53.7)
Smoking	
Present	65 (23.1)
Absent	216 (76.9)
Duration of symptoms	
≤6 hours	130 (46.3)
>6 hours	151 (53.7)
Family history of Ischemic heart disease	
Present	106 (37.7)
Absent	175 (62.3)
Thrombolytic therapy	
Given	194 (69)
Not given	87 (31)
Left ventricular thrombus formation	
Present	65 (23.1)
Absent	216 (76.9)

Our results showed a significant association of LVT formation with age (p = 0.039), hypertension (p: <0.001), symptoms duration (p: <0.01), family history of IHD, and thrombolytic therapy (p: <0.001), as presented in Table 2.

**Table 2:** Association of left ventricular thrombus formation with clinical features and comorbidities Chi-square test was applied; p-value of ≤0.05 considered statistically significant *P-value significant **P-value not significant

Characteristic	N (%)		P-value
	Present	Absent	
Gender			
Male	44 (67.7)	140 (64.8)	0.766**
Female	21 (32.3)	76 (35.2)	
Age group			
≤50 years	11 (16.9)	66 (30.6)	0.039*
>50 years	54 (83.1)	150 (69.4)	
Residence			
Rural	21 (32.3)	85 (39.4)	0.381**
Urban	44 (67.7)	131 (60.6)	
Diabetes mellitus			
Present	22 (33.8)	65 (30)	0.646**
Absent	43 (66.2)	151 (70)	
Hypertension			
Present	43 (66.1)	87 (40.3)	<0.001*
Absent	22 (33.9)	129 (59.7)	
Smoking			
Present	11 (16.9)	54 (25)	0.240**
Absent	54 (83.1)	162 (75)	
Duration of symptoms			
≤6 hours	11 (16.9)	119 (55.1)	<0.001*
>6 hours	54 (83.1)	97 (44.9)	
Family history of Ischemic heart disease			
Present	10 (15.4)	96 (44.4)	<0.001*
Absent	55 (84.6)	120 (55.6)	
Thrombolytic therapy			
Given	11 (16.9)	183 (84.7)	<0.001*
Not given	54 (83.1)	33 (15.3)	

## Discussion

LVT is a known sequel of AMI owing to stasis of blood over damaged heart muscles, which can lead to systemic embolization resulting in a stroke. Early reperfusion is the most effective treatment modality in such circumstances while therapeutic anticoagulation is useful in preventing the risk of thrombosis and embolization [10].

We found LVT formation in 65 (23.1%) of our study subjects, of which 11 (16.9%) were thrombolyzed patients while 54 (83.1%) were non-thrombolyzed. Our findings vary slightly from those of other loco-regional studies. A study conducted by Zia et al. has reported an LVT incidence of 15.4% in patients with AWMI. While LVT was found in 5.4% of the patients thrombolyzed with streptokinase, it was present in 30.4% of those who were not thrombolyzed [9]. Another study from Norway by Solheim et al. has reported similar results, showing a high prevalence of LVT in non-thrombolyzed patients [11].

We found a male predominance (65.9%) in our studied patient population. The male predominance with respect to AMI has been well documented in the literature. Loco-regional literature has shown male gender predominance ranging from 65% to as high as 80% in a few studies [9,12,13]. Our study results were consistent with these findings.

The mean duration of symptoms was 7.25 ± 3.82 hours, and 151 (53.7%) patients had a duration of symptoms for more than six hours. Family history was positive in 106 (37.7%), while thrombolytic therapy was given for 194 (69.0%) of our study cases. The mean age of the patients was 55.54 ± 7.26 years (range: 40-65 years). The mean age of the male patients was noted to be 55.51 ± 8.22 years, while that of female patients was 55.60 ± 4.98 years (p=0.924). Most of the patients (204, 72.6%) were more than 50 years of age. The mean age in other regional studies was reported to range from 53 to 55 years [9,12,13], which was consistent with our data.

Our study has a few limitations. Firstly, this was a single-institution study; however, the institute has a high influx of patients from both the rural and urban areas, and hence the patient population was quite representative of the country's general population. Secondly, we did not engage in a long-term follow-up of the patients to evaluate other long-term complications of AMI in the studied population.

## Conclusions

In our study, we found a high frequency of LVT formation among patients with AWMI who have not undergone thrombolytic therapy. We also observed a significant association of LVT with advanced age, hypertension, and other comorbidities. Early presentation to the hospital and thrombolysis reduce the risk of developing LVT, which in turn can bring down morbidity and mortality in such patients.
